# Acute Tubulointerstitial Nephritis and Secondary Renal Amyloidosis: A Rare Complication of Atezolizumab

**DOI:** 10.7759/cureus.49533

**Published:** 2023-11-27

**Authors:** Naqib Ullah, Sameen Bin Naeem, Mussadique Ali Jhatial, Muhammad Awais Majeed, Saad M Saeed, Shakeel Muzaffar, Maryam Imran, Mudassar Hussain, Samir Fasih

**Affiliations:** 1 Medical Oncology, Shaukat Khanum Memorial Cancer Hospital and Research Centre, Lahore, PAK; 2 Oncology, Shaukat Khanum Memorial Cancer Hospital and Research Centre, Lahore, PAK; 3 Pathology, Shaukat Khanum Memorial Cancer Hospital and Research Centre, Lahore, PAK; 4 Histopathology, Shaukat Khanum Memorial Cancer Hospital and Research Centre, Lahore, PAK; 5 Medical Oncology, Shaukat Khanum Memorial Cancer Hospital and Research Centre, lahore, PAK

**Keywords:** proteinuria, acute interstitial nephritis, secondary amyloidosis, atezolizumab, pembrolizumab, immune check point inhibitors (icis), non-small cell lung cancer (nsclc)

## Abstract

Lung cancer is the second most common malignancy in both genders and the most common cause of cancer-related deaths worldwide. Broadly, lung cancer is divided into two types: small-cell lung cancer (SCLC) and non-small cell lung cancer (NSCLC). Non-small cell lung cancer accounts for 85% of the diagnoses of lung cancer. It is necessary to check for any targetable mutations, which can help in deciding the treatment plan for the patients. The patient we are reporting is a 70-year-old male with multiple co-morbidities diagnosed with non-small cell carcinoma, favoring adenocarcinoma on histopathology. He was started on Atezolizumab/Bevacizumab/Carboplatin/Paclitaxel (ABCP). He was switched to maintenance Atezolizumab/Bevacizumab after four cycles due to poor tolerance to carboplatin and paclitaxel. The patient presented with neutropenic colitis and acute kidney injury (AKI), requiring admission. workup revealed nephrotic range proteinuria with a high urinary albumin-to-creatinine ratio. He underwent a renal biopsy to ascertain the cause of his proteinuria, which showed marked acute and chronic tubulo-interstitial nephritis (TIN), amyloidosis, and global glomerulosclerosis. Secondary (AA) amyloidosis is characterized by the extracellular deposition of misfolded proteins. Although interstitial nephritis is a reported side effect of immune checkpoint inhibitors, AA amyloidosis is a rarer side effect. So, to determine the exact cause and early therapeutic intervention in immune checkpoint inhibitor-related kidney injury, large retrospective or prospective studies should be done.

## Introduction

Lung cancer is the second most common malignancy in both genders' incidence-wise; however, it is the most common cause of cancer-related deaths worldwide [1]. Broadly, lung cancer is divided into two types: small-cell lung cancer (SCLC) and non-small-cell lung cancer (NSCLC). Non-small cell lung cancer accounts for 85% of the diagnoses of lung cancer [2] and can further be sub-classified into four different histological subtypes, with adenocarcinoma being the most common subtype [2]. Treatment of NSCLC depends on the patient’s performance status, comorbidities, tumor stage, and the molecular nature of the disease. Patients with stages I to III are treated with curative intent, which includes surgery, chemotherapy, radiation therapy, or a combined approach [2]. It is necessary to check for any targetable mutations that can help in deciding the treatment of patients with stage IV NSCLC and those with early-stage disease who may require adjuvant tyrosine kinase inhibitor therapy after undergoing curative resection [3]. In the absence of targetable driver mutations, anti-PD-1 or anti-PD-L1 agents (immune checkpoint inhibitors (ICIs), pembrolizumab, and atezolizumab, respectively) can be incorporated into the treatment plan if programmed death ligand 1 (PD-L1) expression is more than 1%. Atezolizumab can also be used in an adjuvant setting in combination with chemotherapy, as reported in the IM Power 150 trial [3]. As far as the safety profile is concerned, most reported immune-related adverse events associated with ICIs include diarrhea, pneumonitis, and hepatitis. 

Here, we report a case of a 70-year-old male who received adjuvant Atezolizumab and developed nephrotic range proteinuria, which eventually was proven to be drug-induced secondary amyloidosis.

## Case presentation

The patient we are reporting is a 70-year-old ex-smoker male with a history of 25 pack years of smoking and comorbidities of hypertension and ischemic heart disease, for the latter of which he underwent percutaneous coronary intervention. He presented to the clinic with a history of hemoptysis for three months. A computed tomography (CT) scan of the thorax was performed, which showed a well-circumscribed lesion in the upper lobe of the right lung along with mediastinal lymphadenopathy. A positron emission tomography (PET) scan showed an upper lobe lesion in the right lung and metabolically avid right hilar and right para-tracheal lymph nodes. His disease was radiologically staged as cT3N2M0 (Stage III-B). A CT-guided biopsy of the lung lesion was performed, which revealed non-small cell carcinoma favoring adenocarcinoma on histopathology. Immunohistochemistry showed positive cytokeratin, TTF1 was focally positive, and p40 and CD56 were negative. Polymerase chain reaction (PCR) for epidermal growth factor receptor (EGFR) did not detect any mutation. Fluorescence in situ hybridization (FISH) was also negative for ALK gene rearrangement. Immunohistochemistry for PDL1 clone SP142 was positive for more than 50% TPS (total proportion score).

**Figure 1:**
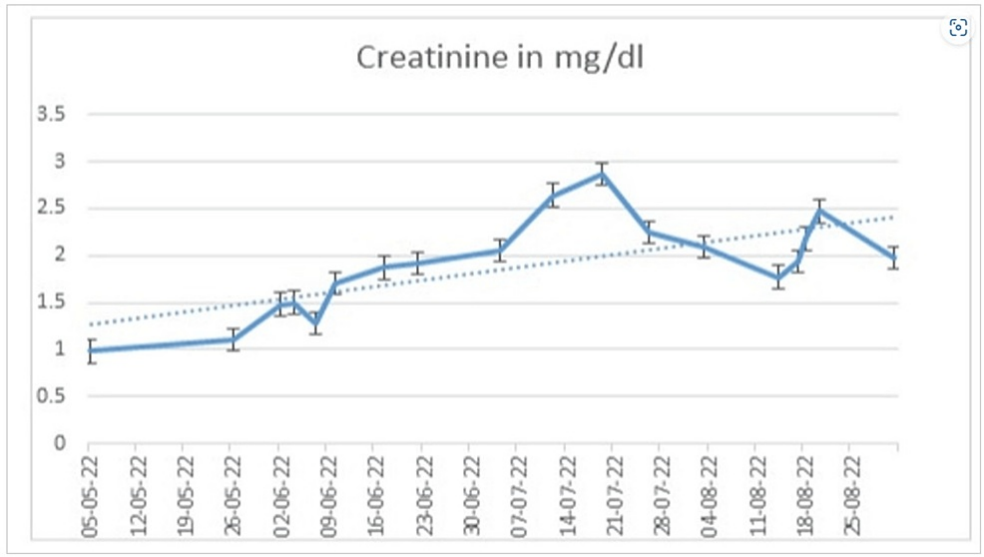
Showing the creatinine trend.

The case was discussed in a multidisciplinary meeting; the patient underwent upfront thoracotomy, leading to a right lung upper lobe lobectomy and mediastinal staging, resulting in pathological stage T3N0. A PET scan performed after surgery showed interval progression with hyper-metabolic pleural-based nodules, nodules in the right lung middle lobe and horizontal fissure, right para-tracheal and internal mammary lymphadenopathy, and bilateral adrenal and bony metastases. He was started on Atezolizumab 1200 mg, Bevacizumab 15 mg/kg, carboplatin AUC 6, and Paclitaxel 200 mg/m2 (ABCP). A restaging PET scan after four cycles of ABCP showed a partial response, with no avidity in pleural nodules, mediastinal nodes, or bony metastases. Adrenal glands also showed responses, with one of the glands showing some increase in size due to necrosis, but avidity in both glands decreased overall. The patient, however, had poor tolerance to chemotherapy with grade II to grade III gastrointestinal side effects, so he was switched to maintenance Atezolizumab/Bevacizumab (AB). After four cycles of maintenance of AB, a PET/CT scan showed interval progression in the left adrenal gland lesion. The left adrenal gland was radiated, and the treatment regimen was switched to Atezolizumab/Pemetrexed (AP). After four cycles of AP, a PET scan showed interval regression in the size and metabolic activity of the left adrenal mass. However, after the 5th cycle of AP, the patient presented with neutropenic colitis and acute kidney injury (AKI), requiring admission. His renal functions continued to deteriorate gradually despite holding chemo-immunotherapy (CIT), as mentioned in Figure 1. 

An interval-staging CT scan was performed after three cycles, which showed stable postsurgical changes in the right lung upper lobe lobectomy without local recurrence or metastatic nodules, along with stable bilateral necrotic adrenal metastases. Further workup for AKI revealed nephrotic range proteinuria with a urine albumin to creatinine ratio of 7915 mg/g (reference < 17 mg/g) and a urine protein to creatinine ratio of 15.54 mg/mg (reference < 0.11 mg/mg). Serum protein electrophoresis was consistent with monoclonal band proteins of 2.1 grams/liter in the beta region, but the Kappa/Lambda ratio was in the normal range (1.55 grams/liter; reference: 1.17-2.93 grams/liter). 

**Figure 2:**
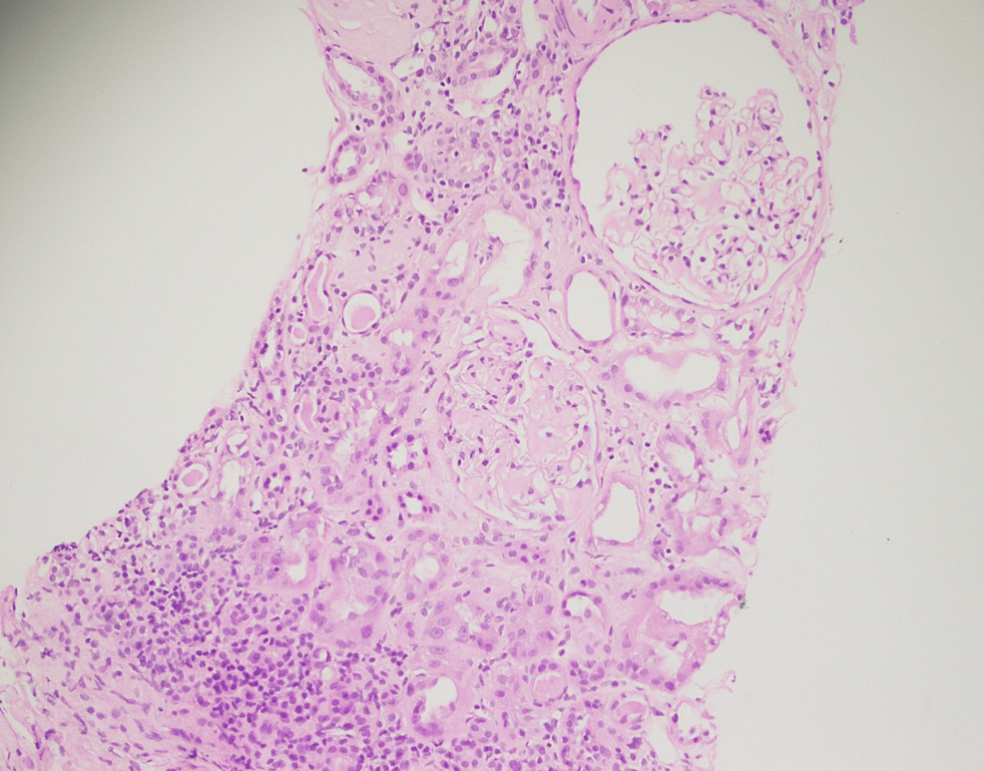
This photomicrograph shows marked acute and chronic interstitial inflammation (lymphocytes, plasma cells, neutrophils, and a few eosinophils), moderate interstitial fibrosis, and tubular atrophy. Arteries and arterioles show hyalinosis.

**Figure 3:**
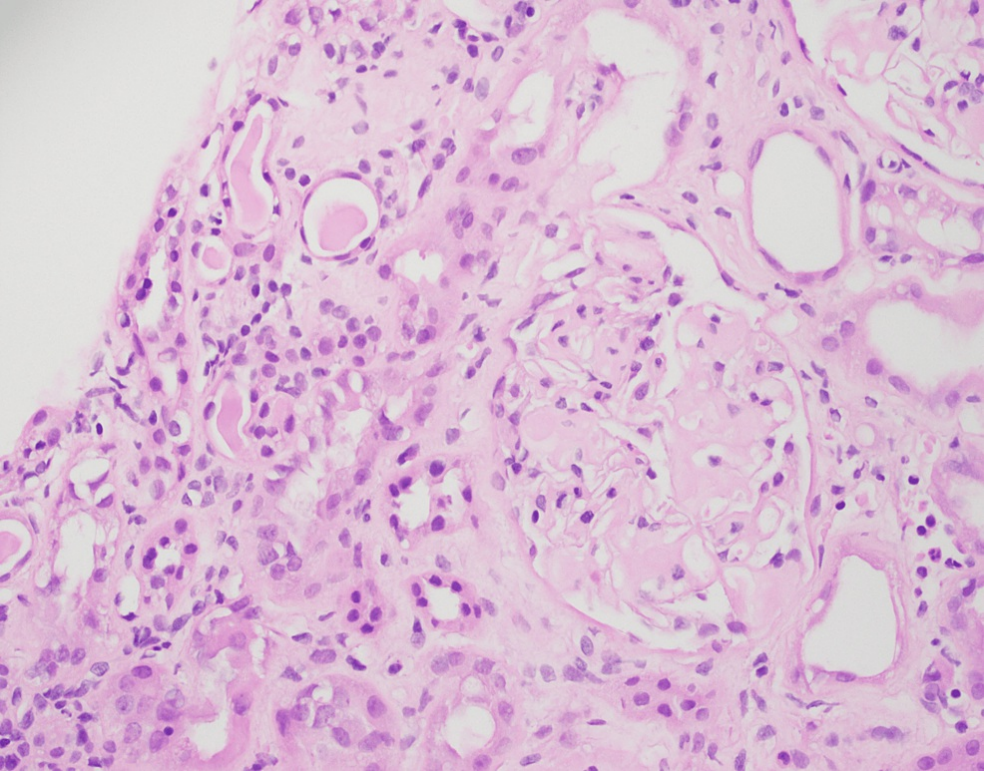
Glomerulus showing eosinophilic mesangial hyalinized deposits. The basement membrane is thickened with hyalinized deposits.

**Figure 4:**
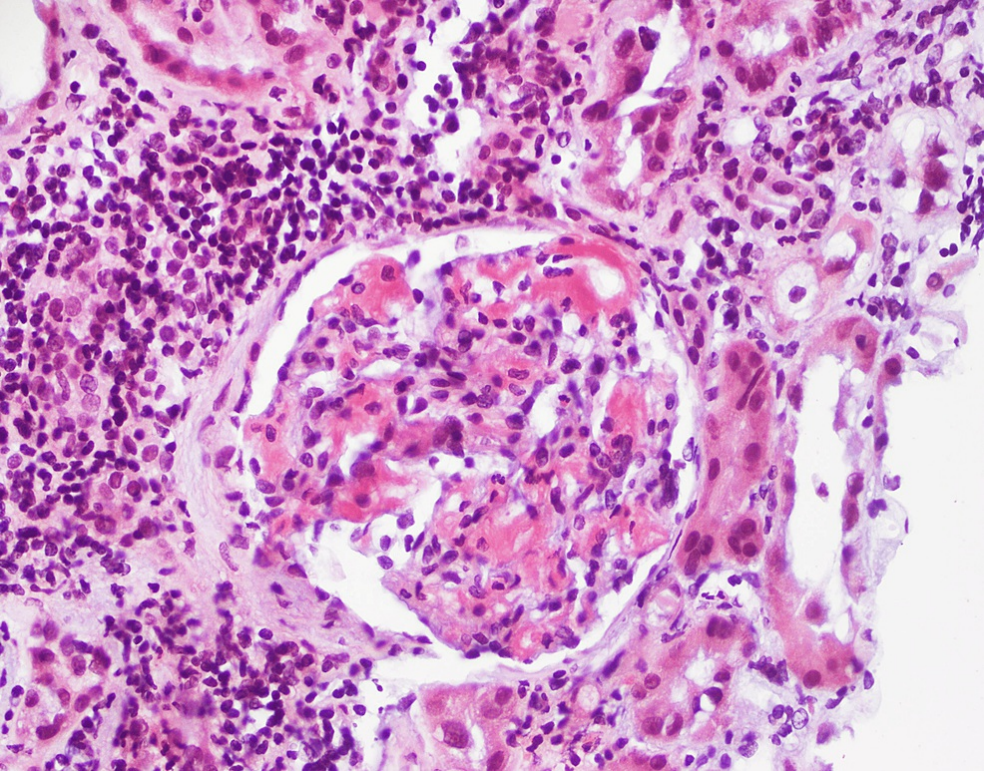
Congo red stain; arrows showing stained hyalinized deposits in mesangium.

**Figure 5:**
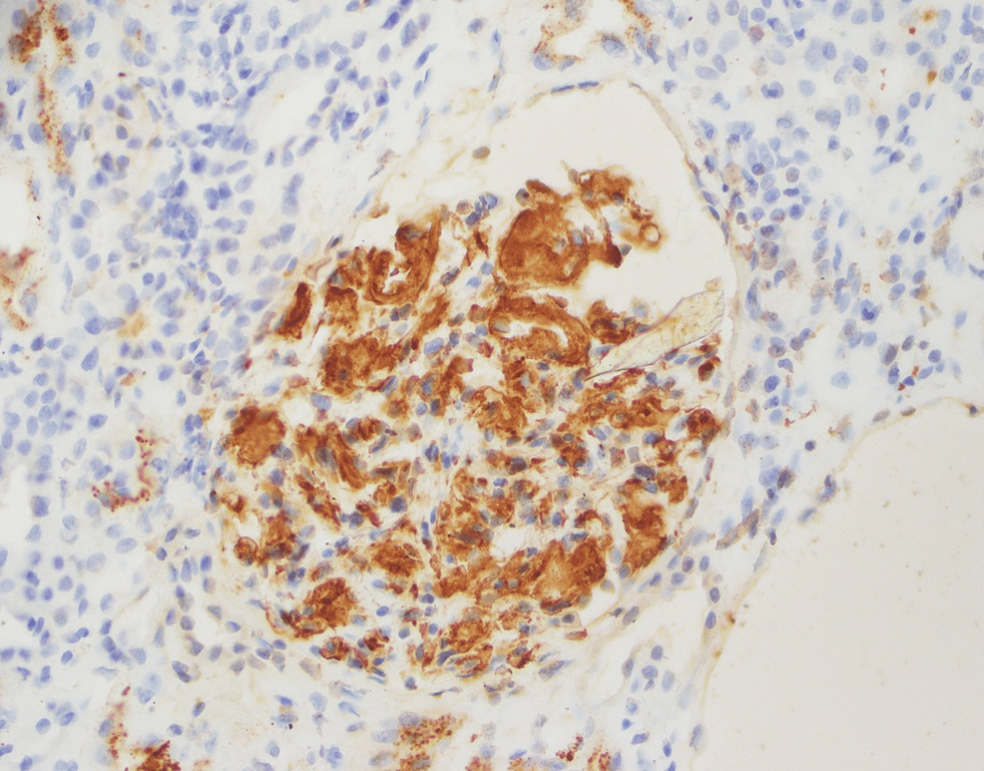
Amyloid A, demonstrating the mesangial deposits.

He underwent a renal biopsy to ascertain the cause of his proteinuria, which showed marked acute and chronic tubulo-interstitial nephritis (TIN), amyloidosis, and global glomerulosclerosis (Figures 2-5). 

The patient started on Prednisolone 1 mg/kg; creatinine came down from 2.8 mg/dl to 1.7 mg/dl after four weeks of steroid therapy, although he continued to have albuminuria. He gradually developed generalized anasarca, which kept on worsening; besides, he developed ESBL *Escherichia coli septicemia*, leading to multi-organ failure and, later, death.

## Discussion

Our patient was an elderly male with PDL-1-positive adenocarcinoma of the lung, stage IIIB, treated with CIT. He had a particularly good treatment response for almost a year. However, he later developed secondary renal AA amyloidosis and tubulointerstitial nephritis, which is a very rare side effect of Atezolizumab, and only a few case reports/case series have documented this side effect [4,5].

Secondary (AA) amyloidosis is characterized by the extracellular deposition of misfolded proteins. AA amyloidosis can occur in various circumstances, including chronic inflammatory disorders, rheumatoid arthritis, and ankylosing spondylitis. Inflammatory bowel disease, chronic infections like tuberculosis, and neoplasms like renal cell carcinoma and lymphoma.

The incidence of AA amyloidosis varies from 1-2 cases per million but is now decreasing with a prevalence of about 5% to 10% [6,7]. In Western countries, the incidence of AA amyloidosis is decreasing due to the low incidence of chronic infections and better treatment for autoimmune diseases; it is now less common than amyloid light-chain (AL) or wild-type transthyretin (senile) amyloidosis. In amyloidosis, intermediate SAA (serum amyloid A) products get aggregated into protofilaments. The kidney is the major involved organ, with proteinuria as the first clinical manifestation. It is diagnosed on a renal biopsy, and the extent of renal damage defines the prognosis. Targeted anti-inflammatory treatment aims to normalize SAA levels and achieve sustained response SAA levels.

Interstitial nephritis is a reported side effect of ICI-related kidney injury, but AA amyloidosis is a rarer side effect. Only a few case reports and a case series have reported this side effect [4]. Data suggests that immune checkpoint inhibitor-related kidney injury may occur as late as 12 months, as in our case [5,8].​​​​​​​

Though pemetrexed can cause renal injury, to the best of our knowledge, no case of AA amyloid has been reported. Available reports suggest that there is PDL-1 expression in the renal tubular epithelium of patients who develop ICI-related kidney injury, and the intensity of expression is related to the severity of renal injury. Treatment of ICI-related kidney injury is the withdrawal of immunotherapy in combination with immunosuppression, leading to improvement in renal functions and a decrease in urinary protein excretion. The extent of inflammation and sclerosis on biopsy determines the recovery, with severe inflammation and sclerosis associated with a poor response to immunosuppression [9].

So, large retrospective or prospective studies should be done to determine the exact cause and role of early therapeutic intervention in immune checkpoint inhibitor-related kidney injury. 

## Conclusions

Immune checkpoint inhibitors play an important role in lung cancer. Interstitial nephritis is a reported side effect of ICI-related kidney injury, but AA amyloidosis is a rarer side effect that needs early diagnosis and treatment. Only a few case reports and a case series have reported this side effect. More reports in the future are required to prove the association of this side effect with Atezolizumab and to determine how to treat it.
